# Empowering Future Healthcare Providers: A Pilot Educational Program for Advance Care Planning in Rural Communities

**DOI:** 10.1093/geroni/igaf122.1420

**Published:** 2025-12-31

**Authors:** Raven Weaver, Cory Bolkan, Amanda Lamp, Kaela Kyaw, Katelyn Costanza

**Affiliations:** Washington State University, Pullman, Washington, United States; Washington State University, Vancouver, Washington, United States; Washington State University, Spokane, Washington, United States; Washington State University, Spokane, Washington, United States; Washington State University, Spokane, Washington, United States

## Abstract

Aging and end-of-life knowledge are critical for healthcare providers, as the majority of high healthcare utilizers are middle-aged or older adult patients. Given population aging, it is essential that healthcare professionals are skilled in facilitating advance care planning (ACP) discussions. Further, Medicare covers ACP services, yet many providers still do not bill for them, citing insufficient preparation for facilitating ACP discussions with patients. To address this gap, our pilot educational project engages and trains interprofessional health students as facilitators of informal community-based ACP discussions in rural settings, where barriers such as geographic isolation, provider shortages, and limited awareness of end-of-life options are significant. Using experiential and competency-based learning frameworks, we trained student volunteers (n = 8) to conduct three community-based ACP events in underserved rural communities. We report on the process evaluation of developing the training, implementing community events, and how we employed a community-based participatory research framework to promote equitable and culturally sensitive ACP. As the project continues, we will also evaluate the overall effectiveness in improving ACP engagement and preparedness via surveys of community participants, student competencies (e.g., communication skills, empathy), and students’ focus-group interviews. Ultimately, this initiative seeks to enhance end-of-life curricula/training for interprofessional health students, empower future healthcare providers with essential ACP communication skills, improve access to ACP in rural areas by scaling up this program for wider dissemination, and address healthcare disparities through patient-centered, community-aligned care.

